# Recombinant expression of beak and feather disease virus capsid protein and assembly of virus-like particles in *Nicotiana benthamiana*

**DOI:** 10.1186/s12985-017-0847-9

**Published:** 2017-09-11

**Authors:** Guy L. Regnard, Edward P. Rybicki, Inga I. Hitzeroth

**Affiliations:** 1 Biopharming Research Unit, Department of Molecular and Cell Biology, Faculty of Science, University of Cape Town, Rondebosch 7701, Cape Town, South Africa; 2 Institute of Infectious Disease and Molecular Medicine, Faculty of Health Sciences, University of Cape Town, Observatory 7925, Cape Town, South Africa

**Keywords:** Plant-made pharmaceutical, Beak and feather disease virus, Virus-like particle, Circovirus, Subunit vaccine

## Abstract

**Background:**

Beak and feather disease virus (BFDV) is an important disease causing agent affecting psittacines. BFDV is highly infectious and can present as acute, chronic or subclinical disease. The virus causes immunodeficiency and is often associated with secondary infections. No commercial vaccine is available and yields of recombinant BFDV capsid protein (CP) expressed in insect cells and bacteria are yet to be seen as commercially viable, although both systems produced BFDV CP that could successfully assemble into virus-like particles (VLPs). Plants as expression systems are increasingly becoming favourable for the production of region-specific and niche market products. The aim of this study was to investigate the formation and potential for purification of BFDV VLPs in *Nicotiana benthamiana*.

**Methods:**

The BFDV CP was transiently expressed in *N. benthamiana* using an *Agrobacterium*-mediated system and plant expression vectors that included a bean yellow dwarf virus (BeYDV)-based replicating DNA vector. Plant-produced BFDV CP was detected using immunoblotting. VLPs were purified using sucrose cushion and CsCl density gradient centrifugation and visualised using transmission electron microscopy.

**Results:**

In this study we demonstrate that the BFDV CP can be successfully expressed in *N. benthamiana*, albeit at relatively low yield. Using a purification strategy based on centrifugation we demonstrated that the expressed CP can self-assemble into VLPs that can be detected using electron microscopy. These plant-produced BFDV VLPs resemble those produced in established recombinant expression systems and infectious virions. It is possible that the VLPs are spontaneously incorporating amplicon DNA produced from the replicating BeYDV plant vector.

**Conclusions:**

This is the first report of plant-made full-length BFDV CP assembling into VLPs. The putative pseudovirions could be used to further the efficacy of vaccines against BFDV.

## Background

Beak and feather disease virus (BFDV; family *Circoviridae*, genus *Circovirus*) is one of the most common disease agents to infect psittacines [1]. The virus infects both wild and captive birds, and has been detected in at least 10% of psittacine species [2–4]. It is the causative agent of psittacine beak and feather disease (PBFD), which can present in three forms. The acute form of the disease has a high mortality rate and is mainly seen in young and neonatal birds [5, 6]. By far the most reported form of the disease is the chronic form, which primarily affects adult birds [7]. Symptoms of chronic disease are the characteristic beak and feather abnormalities associated with PBFD [8]. Birds that are infected with BFDV become immunocompromised, which is the primary cause of chronic infection and persistent excretion of the virus in diseased birds [9, 10]. The third form of the disease is subclinical infection: this form presents the greatest risk for disease spread, as the infected birds continue to shed virus at low concentrations and show no symptoms of infection [11, 12].

A commercial vaccine for BFDV is currently unavailable, and treatment of PBFD is principally palliative and supportive [13–15]. Eradication of the disease is unlikely due to the prevalence of infection and the stability of the virus [16]. A vaccine against BFDV would therefore be desirable, especially due to the presence of subclinical infections that may complicate biocontrol efforts, and since the disease progression varies. The likelihood that a single-strain vaccine would be successful is high, due to the absence of obvious serotypes which indicates conservation of major epitopes between isolates: there is therefore a high probability of cross-isolate protection [17, 18].

**Fig. 1:**
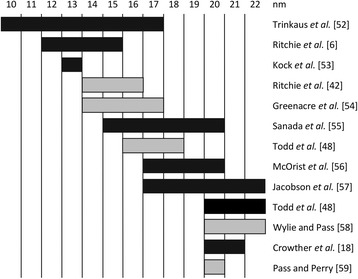
Summary of the BFDV virion size as reported by various authors. Sizes were determined from electron micrographs of particles stained with either uranyl acetate (black) or phosphotungstic acid (grey) Trinkaus*,* et al. [59], Ritchie*,* et al. [8], Kock*,* et al. [60], Ritchie*,* et al. [49], Ritchie*,* et al. [49], Greenacre*,* et al. [61], Sanada*,* et al. [62], Todd*,* et al. [55], McOrist*,* et al. [63], Jacobson*,* et al. [64], Todd*,* et al. [55], Wylie and Pass [65], Crowther*,* et al. [20], Pass and Perry [66]

The virions of BFDV consist of icosahedral capsids, containing a 2-kb circular ssDNA genome that encodes two genes: these are the capsid gene (*cp*), which produces the CP, and the replication associated protein (*rep*)*,* which produces Rep [19–21]. The literature reports the size of the virion to be between 14 and 20 nm in diameter, with the smallest reported size being 10 nm and the largest being 22 nm (Fig. 1). The range of virion sizes appeared to be independent of whether uranyl acetate or phosphotungstic acid was used during the staining procedure. A three dimensional structure determined by cryoelectron microscopy, however, gives a size of 20.5 nm [20]. The most recent analysis of particle size using electron and atomic force microscopy indicates that the CP can form two distinct assemblies with sizes of 10 nm and 17 nm and in addition this could possibly explain the range of reported values that have been described [22]. The virion is extremely stable in the environment, and horizontal transmission through ingestion of contaminated material is seen as the primary route of infection [16, 23]. The persistence of virus outside of the host and a dearth of available treatments have resulted in considerable research towards developing a vaccine against BFDV.

Initial vaccine research centred on inactivated virus vaccines derived from whole tissue of infected birds, purified from feathers or internal organs [12]. This was problematic, however, as production of purified virus was reliant on sourcing infected birds, which has ethical implications and can be severely limiting in terms of availability of harvestable tissue [10]. Furthermore, problems arising from incomplete inactivation of the purified virus have made an inactivated BFDV vaccine an unattractive option [24]. The virus has not as yet been propagated in tissue culture; therefore, the application of recombinant DNA technologies has been viewed as an alternative to infected whole tissue as a source of virus [25].

Recent work towards expression of recombinant BFDV CP shows great promise, with the CP having been successfully expressed using a number of expression platforms, including bacterial and yeast fermentation, insect cell culture and plants [15, 26–29]. Tissue culture technology has also been applied in the expression of other circovirus CPs, such as porcine circovirus (PCV) and goose circovirus (GoCV) [30, 31]. Use of recombinant PCV CP as a vaccine has been shown to result in a humoral response that can produce sterilising immunity that blocks effective virus infection of the host [32, 33]. Limited studies have been conducted on recombinant BFDV CP; however, Bonne*,* et al. [10] reported that vaccination with insect cell-produced CP failed to elicit sterilising immunity, and instead resulted in a decrease in viraemia, replication and virus shedding. This research indicates that recombinant BFDV CP could be used to effect a response in the psittacine immune system, but that further optimisation of the dosage would be required to improve the strength of the immune response.

One important area of focus for recombinant viral vaccine research has been in the production of virus-like particles (VLPs). These lack viral nucleic acids and are therefore non-infectious, while retaining the same structural characteristics of the infectious virion [34]. VLPs are highly immunogenic and stimulate both the humoral and cellular response pathways even in the absence of adjuvants, and have been successfully applied in humans – both the hepatitis B and human papillomavirus vaccines are VLP-based – as well as in humans and poultry, against influenza [35–39]. The BFDV virion has a *T* = 1 symmetry and is assembled from 60 CP subunits that are arranged into 12 pentamer units [20]. The virion of circoviruses is comprised of repeats of a single protein, making it an attractive target for investigation of the production of VLPs – and these have in fact been demonstrated for PCV and demonstrated for bacterial and insect cell-produced BFDV CPs [22, 40–42].

Recombinant BFDV CP expressed in insect cell culture spontaneously assembles into particles ranging in size from 16 to 22 nm in diameter, which falls within the size range reported for infectious virions (10–22 nm in diameter) [41]. Attempts to produced BFDV VLPs using other recombinant systems have been mixed: for example, expression of the full-length BFDV CP using bacterial fermentation resulted in insoluble protein and low yields [15, 27]. Although the expression of a truncated version was successful in increasing yields, this may hinder the assembly of intact VLPs [27]. Sarker*,* et al. [29] have been able to successfully express the BFDV CP and form VLPs in *E. coli* with the yield an order of magnitude greater than reported in previous studies. Plant-based expression in *N. benthamiana* of BFDV CP by Duvenage*,* et al. [26] included a C-terminal-fused 140 or 255 residue elastin-like polypeptide to improve protein yield and ease of purification. The fusion of the BFDV CP to the peptide tag would potentially affect the ability of the CP to assemble into VLPs, and assembly of the plant-expressed BFDV CP into VLPs was not assessed.

In summary, the development of a vaccine against BFDV has moved away from inactivated virus towards the recombinant expression and purification of BFDV CP. This has been shown to successfully produce a humoral response in vaccinated birds. Insect cell and bacterial fermentation-derived BFDV CP have been reported to assemble into VLPs, but this has yet to be shown for plant systems. The aim of this study, therefore, was to investigate the formation and potential for purification of BFDV VLPs in *N. benthamiana,* through the transient expression of the BFDV CP using, among others, a bean yellow dwarf virus (BeYDV)-based replicating DNA vector.

## Methods

### Molecular cloning

**Fig. 2:**
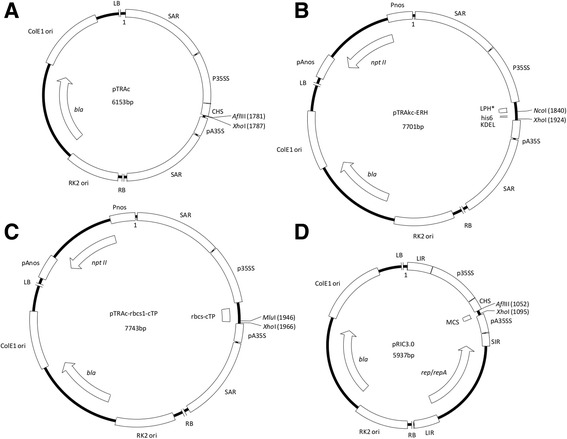
*Agrobacterium* pTRA plant expression vectors: pTRAc (**a**), pTRAkc-ERH (**b**), pTRAkc-rbcs1-cTP (**c**) and pRIC 3.0 (**d**). LB and RB, left and right borders for T-DNA integration; SAR, scaffold attachment region of the tobacco Rb7 gene; P35SS, CaMV 35S promoter with duplicated transcriptional enhancer; CHS, chalcone synthase 5′ untranslated region; pA35S, CaMV 35S polyadenylation signal; RK2 ori, origin of replication for *A. tumefaciens*; *bla*, ampicillin/carbenicillin-resistance *bla* gene; ColE1 ori, origin of replication for *E. coli*; LPH, signal peptide sequence from the murine mAb24 heavy chain; 6xHis, 6 His tag sequence; SEKDEL, ER-retention signal sequence; rbcs1-cTP, chloroplast-transit peptide sequence of a Rubisco small-subunit gene (rbcS1) from *S. tuberosum*; *npt II*, kanamycin-resistance *npt II* gene; Pnos and pAnos, promoter and polyadenylation signal of the nopaline synthase gene; LIR, BeYDV long intergenic region; *rep/repA*, BeYDV *rep/repA* gene; SIR, BeYDV short intergenic region

**Fig. 3:**
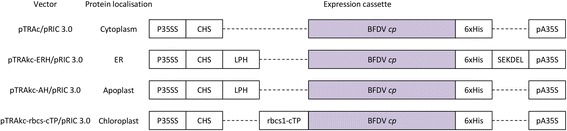
Plant expression and localisation cassettes and *Agrobacterium* plant expression vectors used in this study. BFDV *cp* (purple); P35SS, CaMV 35S promoter with duplicated transcriptional enhancer; CHS, chalcone synthase 5′ untranslated region; pA35S, CaMV 35S polyadenylation signal; 6xHis, 6 His tag sequence; LPH, signal peptide sequence from the murine mAb24 heavy chain; SEKDEL, ER-retention signal sequence; rbcs1-cTP, chloroplast-transit peptide sequence of a Rubisco small-subunit gene (*rbcS1*) from *S. tuberosum*

The BFDV *cp* sequence from isolate BKS1ZA_84 (GenBank accession number GQ165756), isolated by Varsani*,* et al. [43] from a Budgerigar, was synthesised without codon optimisation by Geneart (Germany). The plant expression vectors used in this study were based on the pTRA binary *Agrobacterium tumefaciens* plant expression vector suite which was provided by Prof. Rainer Fischer of the Fraunhofer Institute for Molecular Biology and Applied Ecology in Germany [44]. The vector suite consists of three plant expression vectors targeting protein expression to different locations within the cell: pTRAc (cytoplasm), pTRAkc-ERH (endoplasmic reticulum retained) and pTRAkc-rbcs1-cTP (stromal compartment of the chloroplast) (Fig. 2). Removal of the SEKDEL-encoding sequence for pTRAkc-ERH produces pTRAkc-AH, which allows for the secretion of the recombinant protein to the apoplast. A fourth plant expression vector that was used for the expression of the BFDV CP was pRIC 3.0 (Fig. 2). This vector incorporates sequences of the single-stranded circular DNA genome of BeYDV that allow for vector replication *in planta* [45]. PCR amplification was used to modify the terminal ends of the BFDV *cp* (Table 1)*,* including a C-terminal histidine tag sequence and the introduction of restriction sites to generate a set of non-replicating and replicating constructs with different localisation signals (Fig. 3).

**Table 1:** Primers for PCR amplification used during molecular cloning

Product		Sense primer		Antisense primer	
Description	Size (bp)	Sequence (5′ - 3′)	Cloning site	Sequence (5′ - 3′)	Cloning site
BFDV BKS1ZA_84 cp 6xHis		GTAACGCGTTAGGTACATGTGGGGCACCTCTAAC	*Afl*III/*Mlu*I	GTGATGGTGATGCCCTTCAGTTCTGGGATTATTGG	
	796	GTAACGCGTTAGGTACATGTGGGGCACCTCTAAC	*Afl*III/*Mlu*I	CATCTCGAGCTAGTGATGGTGATGGTGATGCCCTTC	*Xho*I
rbcs1-cTP BFDV BKS1ZA_84 cp 6xHis	959	GGACCATGGCTTCCTCTGTTATTTCCTC	*Nco*I	CATCTCGAGCTAGTGATGGTGATGGTGATGCCCTTC	*Xho*I
BFDV BKS1ZA_84 cp 6xHis SEKDEL		GTAACGCGTTAGGTACATGTGGGGCACCTCTAAC	*Afl*III/*Mlu*I	CTCATCTTTCTCAGAGTGATGGTGATGGTGATGCC	
	814	GTAACGCGTTAGGTACATGTGGGGCACCTCTAAC	*Afl*III/*Mlu*I	CCTCTCGAGCTAGAGCTCATCTTTCTCAGAGTGAT	*Xho*I
LPH BFDV BKS1ZA_84 cp 6xHis SEKDEL	860	GGACCATGGAGTGGAGCTGGATCTTC	*Nco*I	CCTCTCGAGCTAGAGCTCATCTTTCTCAGAGTGAT	*Xho*I
LPH BFDV BKS1ZA_84 cp 6xHis	840	GGACCATGGAGTGGAGCTGGATCTTC	*Nco*I	CATCTCGAGCTAGTGATGGTGATGGTGATGCCCTTC	*Xho*I

Underlined sequence indicates restriction enzyme sites

### *A. tumefaciens*-mediated transient expression

The *A. tumefaciens* GV3101::pMP90RK cells were electroporated using 40–400 ng of recombinant plasmid as described by Maclean*,* et al. [44]. Recombinant clones were selected using agar plates containing kanamycin (30 μg/mL), rifampicin (50 μg/mL) and carbenicillin (50 μg/mL) and incubated at 27° C.

For infiltration, recombinant *A. tumefaciens* GV3101::pMP90RK were grown up overnight at 27° C with agitation in induction medium supplemented with kanamycin (30 μg/mL), rifampicin (50 μg/mL) and carbenicillin (50 μg/mL) [45]. The strain LBA4404 containing pBIN-NSs, provided by Marcel Prins from the Laboratory of Virology, Wageningen in the Netherlands, was supplemented with kanamycin (30 μg/mL), rifampicin (50 μg/mL) and 2 mM MgSO_4_. The addition of MgSO_4_ was to prevent cell clumping during incubation [44]. The NSs protein has been shown to suppress post-transcriptional gene silencing in plants, leading to an increase in transient protein expression [46]. Cells were harvested by centrifugation at 4000 g for 10 min, and resuspended in infiltration medium [45]. The suspensions were diluted to the required absorbance (OD_600_), for expression optimisation studies a range of OD_600_ values were tested, using an Ultrospec™ 10 Cell density meter (Amersham Biosciences, United Kingdom) and incubated at 22° C for 2 h. The *A. tumefaciens* GV3101::pMP90RK suspensions of each expression construct were co-infiltrated with strain LBA4404 containing pBIN-NSs into 6-week-old *N. benthamiana* plants by injecting the suspension into the abaxial spaces using a needleless 1 mL syringe. The plants were maintained in a greenhouse under a 16 h light and 8 h dark photoperiod at light intensity of 60–80 μE/m^2^/s and 22° C.

**Fig. 4:**
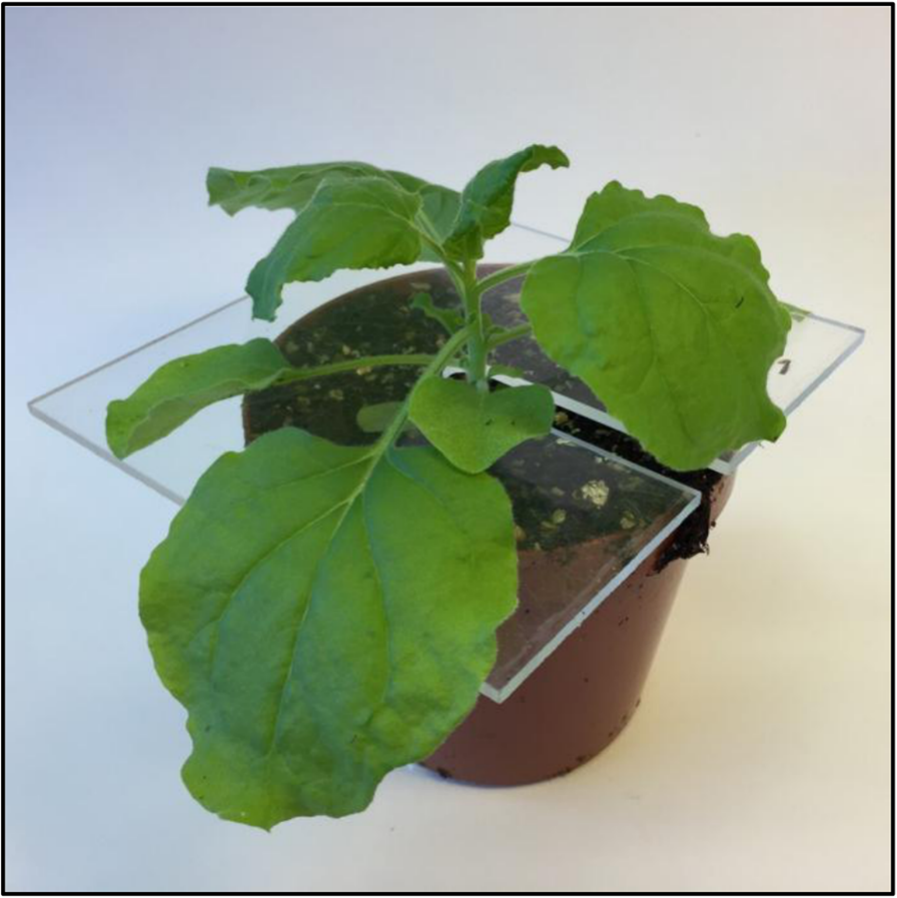
Acrylic seal used to enclose soil during vacuum infiltration of *N. benthamiana*. The acrylic  sheet (130 × 130 mm) prevented soil from upturned plants from falling into the infiltration medium

Vacuum infiltration of *A. tumefaciens* into *N. benthamiana* was performed as described by Maclean*,* et al. [44], with the following modifications. The *Agrobacterium* strains were combined in infiltration medium for a final OD_600_ of 1.00 for strain GV3101::pMP90RK and 0.25 for strain LBA4404 making a total OD_600_ of 1.25. The 6-week-old *N. benthamiana* plants were prepared for inversion into infiltration medium by sealing the base of the plant through the use of a 130 × 130 mm acrylic sheet that contained a 10 mm channel to the centre to allow for the plant stem to be inserted (Fig. 4). This prevented soil from falling into the infiltration medium while the plant was inverted and leaves and stem submerged.

Plants were submerged into the bacterial suspension and subjected to a vacuum of −90 kPa for 5–10 min, with occasional agitation to release trapped air bubbles. The vacuum was released rapidly (approximately 10 kPa/s). In addition, a recombinant expression control, using strain LBA4044 only, was vacuum infiltrated into plants. The plants were grown as described above.

The following controls were included: plant leaf tissue only, plant leaf tissue infiltrated with infiltration medium only, plant leaf tissue infiltrated with strain LBA4404 only, plant leaf tissue infiltrated with strain GV3101::pMP90RK only and plant leaf tissue co-infiltrated with both strains.

### Protein extraction and western blot analysis

For expression optimisation studies leaf discs were harvested at 1, 3, 5 and 7 days post infiltration (DPI). The plant material was prepared into a fine powder using a micro-pestle and liquid nitrogen. One hundred microlitres of extraction buffer (50 mM Tris, 100 mM NaCl, 10% glycerol and 1 mM dithiothreitol at pH 7.5) per leaf disc was added and the leaf material was vortexed. The suspension of leaf tissue was clarified by centrifugation at 1500 g for 3 min and the supernatant representing the crude leaf extract was collected for further analysis. These were incubated at 90° C for 10 min in sample application buffer prepared for analysis by sodium dodecyl sulphate-polyacrylamide gel electrophoresis (SDS-PAGE) [47].

The proteins were resolved on 10% SDS polyacrylamide gels and an equal volume of crude plant extract or equal amount of total soluble protein (TSP) were loaded into each lane. After gel electrophoresis the proteins were transferred onto nitrocellulose membranes at 15 V for 1 h using a Trans-blot®SD semi-dry transfer cell (Bio-Rad, CA, United States of America). The membranes were probed overnight at 4° C with 1:2000 anti-His tag mouse IgG antibody (AbD Serotec, NC, United States of America), and subsequently incubated in a 1:10,000 dilution of anti-mouse IgG (whole molecule) alkaline phosphatase antibody produced in goat affinity isolated antibody (Sigma-Aldrich). Membranes incubated in secondary antibody were washed four times with 1× PBST, with 15 min for each wash. Detection was performed with 5-bromo, 4-chloro, 3-indolylphosphate (BCIP) and nitroblue tetrazolium (NBT) phosphatase substrate (KPL, MD, United States of America).

Vacuum infiltrated plant leaves were harvested on day 3 post-infiltration and approximately 25 g of leaf tissue was used for the extraction of expressed recombinant BFDV CP. Leaves were rinsed in water to remove soil particulates and dried with paper towel to remove excess water, after which the plant material was ground into a fine powder in liquid nitrogen using a mortar and pestle. The plant material was then combined with PBS containing cOmplete™ Mini, EDTA-free protease inhibitor (Roche) using a ratio of 1:2 (*w*/*v*) of plant material to extraction buffer. PBS has previously been used as a buffer for the extraction of BFDV virions from whole tissue of diseased psittacines [48]. The mixture was then homogenised for 5 min at 10000 rpm and 4° C using a T 25 digital ULTRA-TURRAX® (IKA® Works Inc., NC, United States of America). The homogenate was centrifuged at 4000 g for 10 min at 4° C using a JA-14 rotor (Beckman Coulter, CA, United States of America). The supernatant was then filtered through two layers of Miracloth (Merck, Germany) in preparation for sucrose cushion centrifugation.

### Sucrose cushion centrifugation

Sucrose cushion centrifugation was based on the technique for purification PCV-2 capsid particles described by Wu*,* et al. [42]. A 40% *w*/*v* sucrose solution (470.6 mg/mL of solution) was prepared in water and confirmed using a R5000 hand refractometer (Atago, Japan). A 2 mL 40% sucrose cushion was prepared in 5 mL Ultra-Clear™ centrifugation tubes (Beckman Coulter) and layered with supernatant derived from the slow speed centrifugation step. The tubes were centrifuged in a SW 55 Ti rotor (Beckman Coulter) at 40500 rpm (RCF_max_ ≈ 200,000 g) for 6 h at 4° C. The pellet was resuspended in 500 μL of PBS and further analysed by transmission electron microscopy (TEM).

### Transmission electron microscopy

Analysis of BFDV CP particle assembly was done using TEM. Copper grids (mesh size 200) were made hydrophilic by glow discharging at 25 mA for 30 s using a Model 900 SmartSet Cold Stage Controller (Electron Microscopy Sciences, PA, United States of America). The grids were then placed on a 1:100 dilution of the samples for 5 min and then washed three times in sterile water. The grids were negatively stained on uranyl acetate for 10 s and again for a further 20 s and viewed using a Tecnai™ F20 Scanning Transmission Electron Microscope (FEI, OR, United States of America).

### CsCl density gradient centrifugation

CsCl density gradient centrifugation was performed based on a protocol modified from Ritchie*,* et al. [49]. Leaves containing expressed recombinant BFDV CP were extracted as described above using a 50 mM Tris at pH 7.6 buffer at a ratio of 3:1 (*v*/*w*) buffer to plant material. The sample together with a 5 mL sucrose cushion (45% *w*/*v* sucrose solution; 541.1 mg/mL of solution) was centrifuged in a SW 32 Ti rotor (Beckman Coulter) at 32000 rpm (RCF_max_ ≈ 175,000 g) for 2 h at 4° C. The resulting pellet was used for CsCl density gradient fractionation.

The pellets were resuspended in Tris buffer containing CsCl at a density of 1.406 g/cm^3^. CsCl density was confirmed using a R5000 hand refractometer (Atago). The resuspended pellets were centrifuged using a SW 55 Ti rotor (Beckman Coulter) at 48000 rpm (RCF_max_ ≈ 280,000 g) for 20 h at 20° C. The CsCl gradient was fractionated using a Foxy® Jr. Fraction Collector (Teledyne Isco, NE, United States of America). The refractive index for each fraction was determined using the refractometer; 10 μL of each fraction was spotted onto nitrocellulose membrane and western blot analysis was performed as described above. The BFDV CP present on the nitrocellulose membrane was then quantified using a Syngene Gene Genius imaging system and GeneTools software (Synoptics Inc., United Kingdom). In addition to quantifying the BFDV CP in each fraction, a ND-1000 Spectrophotometer (NanoDrop®, DE, United States of America) was used to determine the TSP (Protein A280 module) and total DNA (Nucleic acid module).

Fractions containing the greatest concentration of BFDV CP were pooled and dialysed using dialysis tubing cellulose membrane (Sigma-Aldrich) against 50 mM Tris at pH 7.6. Dialysed pooled fractions were analysed using western blotting as described above.

## Results

### Optimisation of BFDV CP expression in *N. benthamiana*

To determine the best conditions for expression, various densities of *Agrobacterium* suspensions were tested by plant syringe infiltration, and leaves were harvested on days 1, 3, 5 and 7. The optimum optical density for *Agrobacterium* suspension infiltration was determined to be 0.50 and 1.00 for the expression of BFDV CP and 0.25 for the expression of the silencing suppressor NSs, while the optimum day post-infiltration to harvest was determined by western blotting to be day 3 (data not shown). The effect of targeting the full-length BFDV CP to different organelles - chloroplast and ER, and secretion to the apoplast - was assessed together with gene amplification due to the replicating expression vector.

**Fig. 5:**
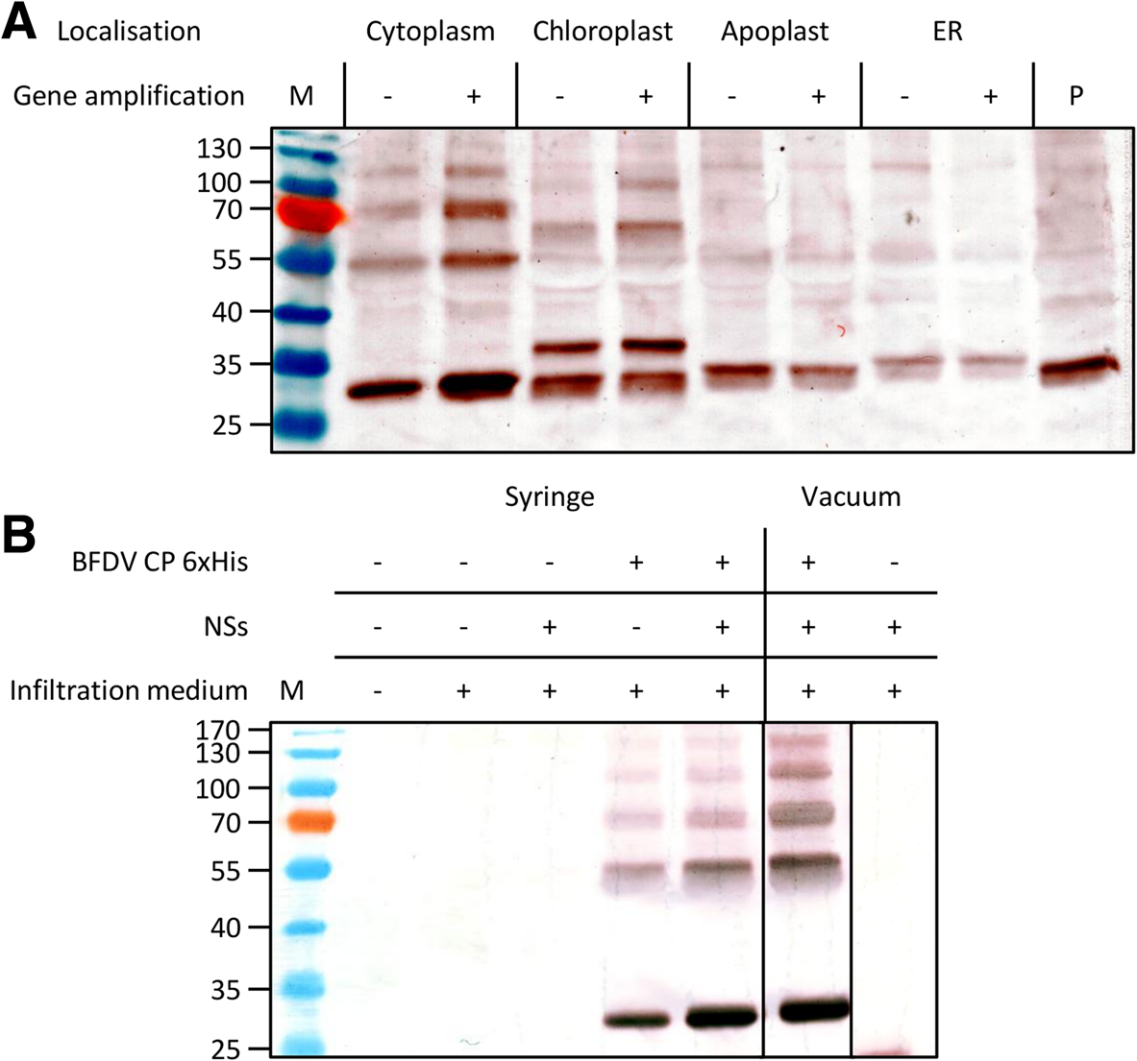
Western blot analysis of BFDV CP expression. **a** Analysis of the effect of targeted BFDV CP localisation and BFDV *cp* gene amplification on expression. Protein was extracted on day 3 from *N. benthamiana* leaves. Expression of the CP in the cytoplasm (30.1 kDa), the chloroplast (36.0 kDa), ER (33.0 kDa) and secretion into the apoplast (32.3 kDa). Expression in the presence (+) or absence (−) DNA amplification of the *cp* was assessed in all cases. An equal amount of TSP was loaded into each lane and the CP was detected using anti-histidine antibody. M – prestained protein marker, P – positive control. **b** Analysis of BFDV CP expression comparing syringe versus vacuum infiltration of *Agrobacterium*. Negative controls were non-infiltrated plant leaves, leaves infiltrated with medium and NSs only. The presence (+) or absence (−) of each component is specified. TSP was extracted from leaf tissue harvested on day 4 post-infiltration. An equal volume of each sample was loaded into the lanes

The CP was expressed by each plant expression vector (Fig. 5a). Additional proteins with higher molecular weights than the CP were detected in the SDS-PAG electropherograms. The cytoplasm was the most favourable location for accumulation, followed by the chloroplast and the ER, while accumulation of CP in the apoplast was the lowest. Gene amplification had a mixed effect on expression: accumulation in the cytoplasm and chloroplasts appeared to be improved; however, accumulation in the ER decreased, and there was no change when the CP was secreted to the apoplast. Overall, expression in the cytoplasm with gene amplification was determined to be the most favourable.

### Vacuum infiltration of *N. benthamiana* for the expression of BFDV CP

A comparison was made between the two methods of infiltration using the replicating pRIC 3.0 vector without a localisation signal (cytoplasm, Fig. 5b): the 30.1 kDa CP was successfully expressed in *N. benthamiana* using both methods. The silencing suppressor evidently improved expression of the CP, as an increase in band intensity was apparent for the CP co-expressed with NSs.

**Fig. 6:**
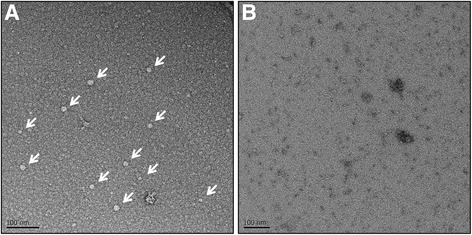
Transmission electron micrographs of the partially purified BFDV VLPs (indicated by white arrows) after sucrose cushion centrifugation. **a** Resuspended pellet from leaf tissue expressing the BFDV CP with C-terminal histidine tag together with the silencing suppressor NSs. **b** Resuspended pellet from leaf tissue expressing only the silencing suppressor NSs which served as a negative control

The pellet deriving from the sucrose cushion centrifugation of clarified plant extract was resuspended and analysed by TEM (Fig. 6). This analysis showed regular particles ranging between 13 and 23 nm in diameter in the pellet deriving from leaf tissue expressing the BFDV CP together with the silencing suppressor NSs (Fig. 6a). The predominant size detected was ~17 nm in diameter. Analysis of the pellet from leaf tissue expressing the silencing suppressor NSs only showed nothing consistent with VLPs (Fig. 6b).

**Fig. 7:**
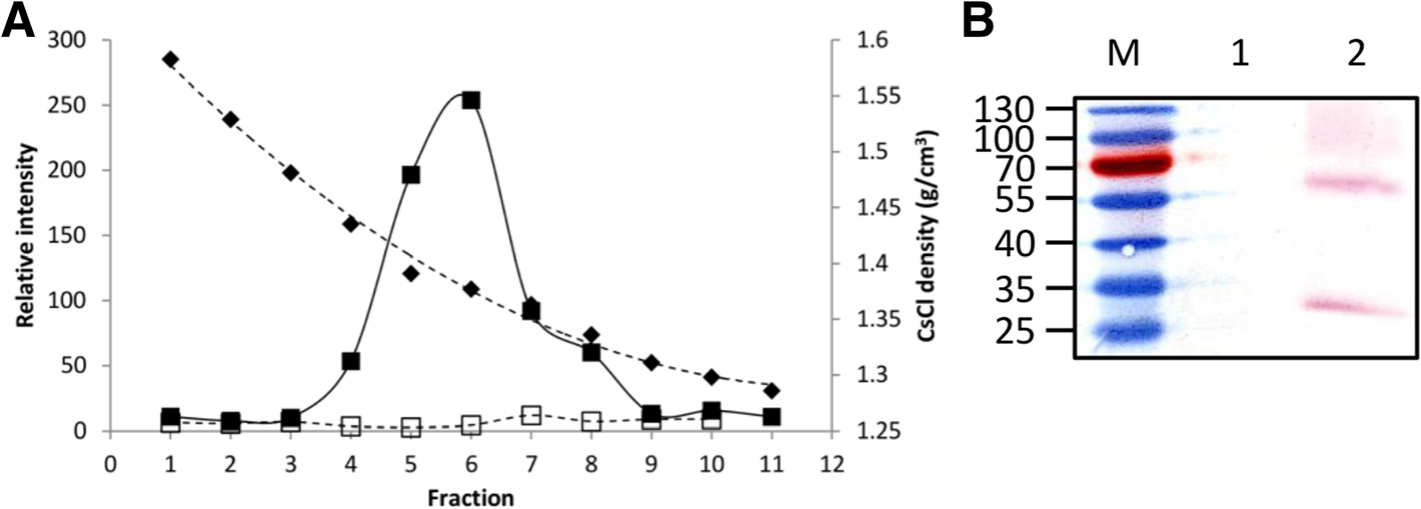
CsCl density gradient centrifugation profile of BFDV CP. **a** The CsCl density (♦) and relative intensity of histidine tagged protein as determined by dot-blot from leaf tissue co-expressing the BFDV CP and NSs silencing suppressor (■) or the NSs silencing suppressor alone (□). **b** Western blot analysis of pooled fractions 5–7 after dialysis. M – Prestained protein marker, 1 – NSs control, 2 – BFDV CP and NSs. An equal volume of each sample was loaded into the lanes and the CP was detected using anti-histidine

The BFDV CP/NSs co-expression and the NSs control pelleted material were further analysed by CsCl density gradient centrifugation. Fractions of the CsCl density gradients were collected for western blotting, and spectrophotometric analysis (Fig. 7): the fractions represent a CsCl density gradient that ranged from 1.29 g/cm^3^ to 1.58 g/cm^3^. Dot blot analysis using anti-histidine antibody on gradient fractions indicated a peak in staining intensity at fraction 6 for samples containing the BFDV CP co-expressed with the NSs silencing suppressor, and a shoulder peak in fraction 8 (Fig. 7a). These peaks were absent in the NSs-only samples**.** The peak fraction 6 for the CP/NSs sample corresponded to a CsCl density of 1.38 g/cm^3^. The shoulder peak (fraction 8) had a CsCl density of 1.34 g/cm^3^. Fractions 5–7 of for each density gradient were pooled, dialysed and analysed by Coomassie blue staining and western blotting of SDS-PAGE gels (Fig. 7b). The BFDV CP was undetectable when gels were stained with Coomassie blue (data not shown). However, bands corresponding to the histidine-tagged 30.1 kDa CP and a protein of approximately 60 kDa were detected in the CP/NSs sample, while no histidine-tagged proteins were detected for the pooled fractions of the NSs silencing suppressor expressed on its own.

**Fig. 8:**
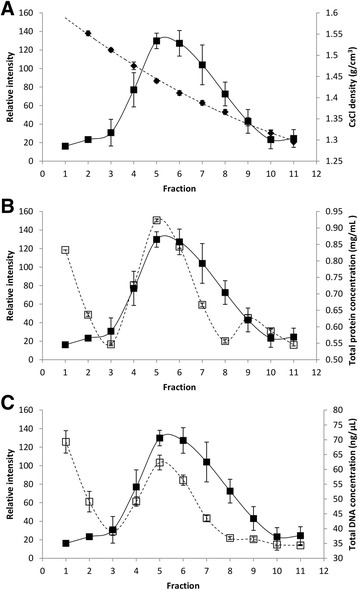
CsCl density gradient centrifugation profiles of BFDV CP, protein and DNA concentration. Comparison of relative intensity of histidine tagged protein as determined by dot-blot from leaf tissue co-expressing the BFDV CP and NSs silencing suppressor (■) with **a** CsCl density (♦), **b** TSP concentration (□) and **c** total DNA concentration (□)

Further CsCl density gradient fraction analysis included spectrophotometry for protein (280 nm) and DNA (260 nm) concentrations. Three BFDV CP/NSs gradients were analysed (Fig. 8) by dot blot analysis using anti-histidine antibody compared to CsCl density, and by TSP and total DNA concentration determination. The CsCl density gradient ranged from 1.29 g/cm^3^ to 1.55 g/cm^3^. Again, a dot blot intensity peak was observed between fraction 5 and 6, corresponding to CsCl densities between 1.41 g/cm^3^ and 1.44 g/cm^3^ (Fig. 8a). The shoulder peak seen in the previous results was absent. A peak in TSP of 0.92 mg/mL was seen in fraction 5 (Fig. 8b). A second smaller peak for TSP was observed in fraction 9 (CsCl density of 1.34 g/cm^3^), while a final peak was present at a density of 1.55 g/cm^3^. Similar peaks were detected for total DNA concentration (Fig. 8c).

## Discussion

The recombinant expression of BFDV CP in *N. benthamiana* was optimised by determining the optimum optical density of *Agrobacterium* at infiltration, the optimum day post infiltration for leaf harvesting, and the most favourable localisation of BFDV CP inside various cellular compartments (Fig. 5a). Localisation in the cytoplasm led to the greatest accumulation of CP, on day 3 PI, when compared to chloroplast, apoplast and ER localisation. This corresponds well with what happens with the CP during viral infection, when CP expressed in the cytoplasm is transported into the nucleus by means of the nuclear localisation signal (NLS) encoded in its sequence [27]. While nuclear localisation was not investigated here, it is possible that presence of the NLS may negatively affect targeting to other cellular compartments: the NLSs are located in the N-terminal region and could compete effectively with other N-terminal signal tags. For example, chloroplast signal-tagging of the CP in this work resulted in the presence of an additional protein band of higher molecular weight: this could result from the CP being transported into the nucleus before the signal tag could be cleaved and CP deposited into the chloroplast. In order to confirm this, fluorescently labelled BFDV CP could be used together with an inverted epifluorescence microscope to trace the localisation of the protein in the plant cell as has been demonstrated using insect tissue culture by Heath*,* et al. [27]. Targeting the CP to the ER, with or without inclusion of the ER retention SEKDEL sequence, resulted in a reduction in CP accumulation. It could be suggested that retention in the ER resulted in the CP being diverted to proteolytic vesicles, which would reduce the overall accumulation of CP in the plant cell. Alternatively, secretion to the apoplast could also result in degradation by abundant proteases in this compartment. While accumulation in the cytoplasm was satisfactory, an additional cellular compartment that could be investigated is the vacuole: Thomas and Walmsley [50] reported the accumulation of human epidermal growth factor was greatest when targeted to the vacuole, when compared to the ER and apoplastic space.

An increase in gene copy number had a variable effect on protein accumulation, dependent on the protein localisation. Gene amplification increased the overall protein accumulation in the cytoplasm and chloroplast, while a decrease/no effect in accumulation was observed in the apoplastic space and ER. As has been previously reported, an increase in gene copy number by means of gene amplification based on BeYDV rolling circle replication (RCR) could result in up to three orders of magnitude increase in gene copy number, with only a marginal increase in protein accumulation [45]. This could be as a result of diversion of cell resources away from transcription and protein translation to DNA replication. Analysis of mRNA transcription from a non-replicating plant expression vector has shown transcript levels to peak at three orders of magnitude higher [51]. Gene amplification would therefore probably compete with transcription for cell resources. A solution to this would be to control the level of gene amplification to much lower levels.

Although the various optimisations increased the expression level of BFDV CP, the protein was undetectable by SDS-PAGE when stained with Coomassie blue. Coomassie blue dyes can detect as few as 25 ng per band for most proteins: this would indicate that the concentration of BFDV CP was below 625 ng/mL. The BFDV CP would represent less than 0.00025% of TSP, and in terms of overall yield of BFDV CP per gram of fresh weight, this would be less than 5 mg/kg. This is similar to recombinant expression levels seen for plant-produced HIV p17 ⁄ p24 of 5 mg/kg [45, 52], but far lower than the >500 mg/kg achieved for HPV-16 L1 protein using the same vector systems [44, 45]. For plant-produced BFDV to be viable, yields approaching at least 50 mg/kg are required to commercial production, as has been reported for influenza VLPs [53].

Vacuum infiltration resulted in the same level of expression as when *Agrobacterium* suspensions are infiltrated using a needleless syringe, and as has previously been demonstrated, the co-expression of the silencing suppressor protein NSs results in an increase in BFDV CP accumulation (Fig. 5b). It could be suggested that the larger proteins detected in this study are potentially CP dimers, trimers and tetramers, and that these could potentially be precursors to the pentameric subunits described by Crowther*,* et al. [20].

TEM analysis of the sucrose cushion pellet revealed negatively stained particles that were circular and featureless (Fig. 6). This description perfectly fits the infectious BFDV particles seen by Crowther*,* et al. [20]. The plant-produced BFDV particles ranged between 13 and 23 nm in diameter, and were similar to putative BFDV VLPs produced in insect cell cultures, which ranged between 16 and 22 nm diameter [41]. The predominant diameter measured was approximately 17 nm, which falls neatly within the 10–22 nm range reported for infectious virions, and is the same diameter as has been reported for the solved atomic structure of BFDV VLPs [22]. These values are similar to insect cell-produced PCV VLPs that averaged 20 nm in diameter, closely resembling the infectious PCV virion that consists of a 1.7-kb genome encapsidated within a 20.5 nm non-enveloped capsid [20]. The addition of a histidine tag on the C-terminus, used for immunodetection, did not appear to negatively affect particle structure; however, the effect on the antigenicity of the protein would need to be addressed before immunogenicity studies.

Fractionation of CsCl gradients of the pellet produced during sucrose cushion centrifugation produced two peaks of BFDV CP. The major peak occurred at an approximate density of 1.38 g/cm^3^, while a shoulder peak at 1.34 g/cm^3^ was also detected (Fig. 7). A similar density of 1.365 g/cm^3^ has been reported for PCV VLPs produced in Tn5 insect cells [54]. The higher observed density could also be the result of the presence of the C-terminal 6xHis tag. The absence of viral DNA has been seen to affect the density of the VLPs. Infectious BFDV virions purified using CsCl have a density ranging between 1.35–1.378 g/cm^3^, while intact VLPs range between 1.215–1.325 g/cm^3^ [41, 48, 55]. A similar decrease in density has been reported for PCV VLPs [40].

To further investigate the purification of BFDV VLPs spectrophotometric analysis of protein and DNA concentrations were performed. The CsCl gradient fractions from material made using the recombinant replicating geminivirus-derived vector revealed coincident peaks corresponding to the protein and DNA (Fig. 8). This suggests that the BFDV CP could be packaging single-stranded amplicon DNA, as the viruses have very similar replication cycles. It could be that the major peak represents VLPs containing plant expression vector replicon DNA generated from RCR, and the shoulder peak could represent empty VLPs. Considering the BeYDV replicon is approximately 3.3-kb it is questionable whether it could be successfully packaged by the BFDV CP. A BeYDV replicon that was similar in size to the 2-kb BFDV genome would be more suitable for encapsidation. Plant viral CPs have been shown to effectively and spontaneously package nucleic acids of mammalian viruses [56]. These pseudovirions have then been shown to release their nucleic acids into the cytoplasm of mammalian cells. This technology has also been used successfully using a plant-produced bamboo mosaic virus particles containing infectious bursal disease virus antigens for the immunisation of chickens [57]. Plant-produced CP derived from circoviruses could similarly be used to package nucleic acids that have been replicated *in planta* using the BeYDV replication system. These pseudovirions could then potentially be used to create a potent vaccine capable of eliciting a strong humoral and cellular response in the target host. This has previously been demonstrated in our group for plant-made human papillomavirus pseudovirions used in neutralisations assays [58].

## Conclusions

In conclusion, BFDV CP has been shown to be successfully expressed in *N. benthamiana* via syringe or via vacuum infiltration. It has also been shown to self-assemble into VLPs even when fused with a C-terminal histidine tag that can be detected using electron microscopy and purified using CsCl centrifugation. These plant-produced BFDV VLPs resemble those produced in insect cells and infectious virions; however the yield was low, as the CP was undetectable when stained with Coomassie. The detection of VLPs in plants allows for refinement of the purification method, possibly by incorporating knowledge of the sedimentation coefficient determined for PCV and CAV to improve isolation by centrifugation. It is possible that the VLPs are spontaneously incorporating amplicon DNA produced from the replicating BeYDV plant vector. The putative pseudovirions could be used to further the efficacy of vaccines against BFDV.

## Abbreviations

BCIP: 5-bromo, 4-chloro, 3-indolylphosphate
BeYDV: bean yellow dwarf virus
BFDV: beak and feather disease virus
*cp*: capsid gene
CP: capsid protein
DPI: days post infiltration
GoCV: goose circovirus
NBT: nitroblue tetrazolium
PBFD: psittacine beak and feather disease
PCV: porcine circovirus
*rep*: replication associated protein
SDS-PAGE: sodium dodecyl sulphate-polyacrylamide gel electrophoresis
TSP: total soluble protein
VLPs: virus-like particles
